# Primary spontaneous pneumothorax: reassessing the role of chest CT in surgical decision-making

**DOI:** 10.1007/s12055-025-02014-6

**Published:** 2025-08-08

**Authors:** Mohammad Abu El Hija, Harbi Khalayleh, Guy Pines

**Affiliations:** 1 Department of Radiology, Kaplan Medical Center, Rehovot, Israel; 2 Department of Surgery A, Kaplan Medical Center, Rehovot, Israel; 3 Department of Thoracic Surgery, Kaplan Medical Center, POB 1, 76100 Rehovot, Israel; 4 Department of Surgery B, Kaplan Medical Center, Rehovot, Israel; 5 Faculty of Medicine, Hebrew University of Jerusalem, Jerusalem, Israel

**Keywords:** Primary spontaneous pneumothorax, Chest imaging, Surgical decision-making

## Abstract

**Purpose:**

Primary spontaneous pneumothorax (PSP) is a common condition managed either conservatively or surgically. This study investigated whether specific chest X-ray or computed tomography (CT) characteristics can predict the need for surgical intervention and clarified the role of each imaging modality in surgical planning.

**Methods:**

We retrospectively reviewed records of all PSP patients at a university hospital from 2013 to 2024. Only patients with available imaging were included in the analysis. Radiographic characteristics, clinical data, and outcomes were collected. Univariate analysis and logistic regression were performed to identify predictors of surgical intervention.

**Results:**

Ninety-one patients were included, with 35 (38.5%) undergoing surgical intervention. Chest X-ray findings did not significantly correlate with surgical decisions (odds ratio (OR) = 0.920, 95% confidence interval (CI) 0.239–3.533, *P* = 0.903). Patients with recurrent pneumothorax were more likely to require surgery (74.3% vs. 25.7%, *P* < 0.0001). Logistic regression revealed that symptom onset 1 day prior to admission increased the likelihood of surgery 4.122-fold compared to same-day onset (95% CI 1.004–19.920, *P* = 0.049). Eighteen patients underwent CT scanning, and their chest X-ray findings closely correlated with CT findings (kappa’s score = 0.944, 95% CI 0.83–1.00).

**Conclusions:**

Chest X-ray findings alone are insufficient predictors of surgical intervention in PSP. Instead, prolonged symptom duration and a history of recurrence are key determinants of surgical need. The strong correlation between X-ray and CT findings suggests that routine preoperative CT may add little value, particularly in young patients. Further prospective studies are needed to validate these results.

**Graphical abstract:**

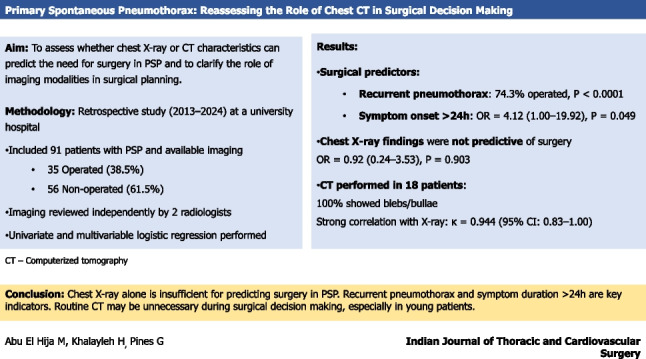

**Supplementary Information:**

The online version contains supplementary material available at 10.1007/s12055-025-02014-6.

## Introduction

Primary spontaneous pneumothorax (PSP) is common and can range from mild discomfort to life-threatening respiratory compromise, with recurrence rates of 25–54% within the first year [1–3]. Surgical intervention is indicated for recurrent or bilateral PSP, tension pneumothorax, significant hemopneumothorax, prolonged air leak, or failure of lung re-expansion, and in high-risk populations such as pilots, scuba divers, pregnant women, residents of remote areas, and elite athletes [4]. Despite established surgical criteria, predicting which patients will require surgery based solely on initial presentation remains challenging.

Chest radiography is the primary diagnostic modality for PSP due to its accessibility, while computed tomography (CT) offers higher sensitivity and specificity by detecting subtle abnormalities and underlying pathology [5]. However, the prognostic value of specific radiographic findings in determining the need for surgical intervention is unclear.

This study aims to assess whether chest X-ray characteristics can predict surgical intervention in PSP patients and to evaluate the routine use of CT in surgical planning.

## Materials and methods

Institutional review board approval was obtained for this study. The records of all patients aged 18–60 who were treated for PSP in a university hospital between 01/01/2013 and 01/01/2024 were reviewed. Inclusion criteria included all patients with PSP, within the specified age range, with imaging available for evaluation. Patients were excluded if they had pneumothorax from other known causes or if imaging was not available for evaluation. Patient demographics, clinical characteristics, and imaging data were collected from electronic medical records. Clinical data included vital signs (systolic and diastolic blood pressure, heart rate, and oxygen saturation), smoking status, duration of symptoms, type of chest drain used (small-bore drains <14 French or larger drains ≥14 French), lung expansion, and rate of improvement. The number of recurrent pneumothorax events before surgery and the cases of surgical intervention were also recorded.

Cohort was divided into two groups: operated group—patients who ultimately underwent a surgical intervention for their PSP; non-operable group included patients managed without definitive surgical intervention. Chest drains were placed in both groups regardless of patients’ assignment.

### Imaging analysis

#### Chest X-rays

All available chest X-rays were reviewed by a single radiologist and independently verified by two other radiologists. X-ray measures included the side of the pneumothorax, size, apical irregularity, excess lucencies, pulmonary collapse, and radiologic signs of tension pneumothorax.

**Fig. 1:**
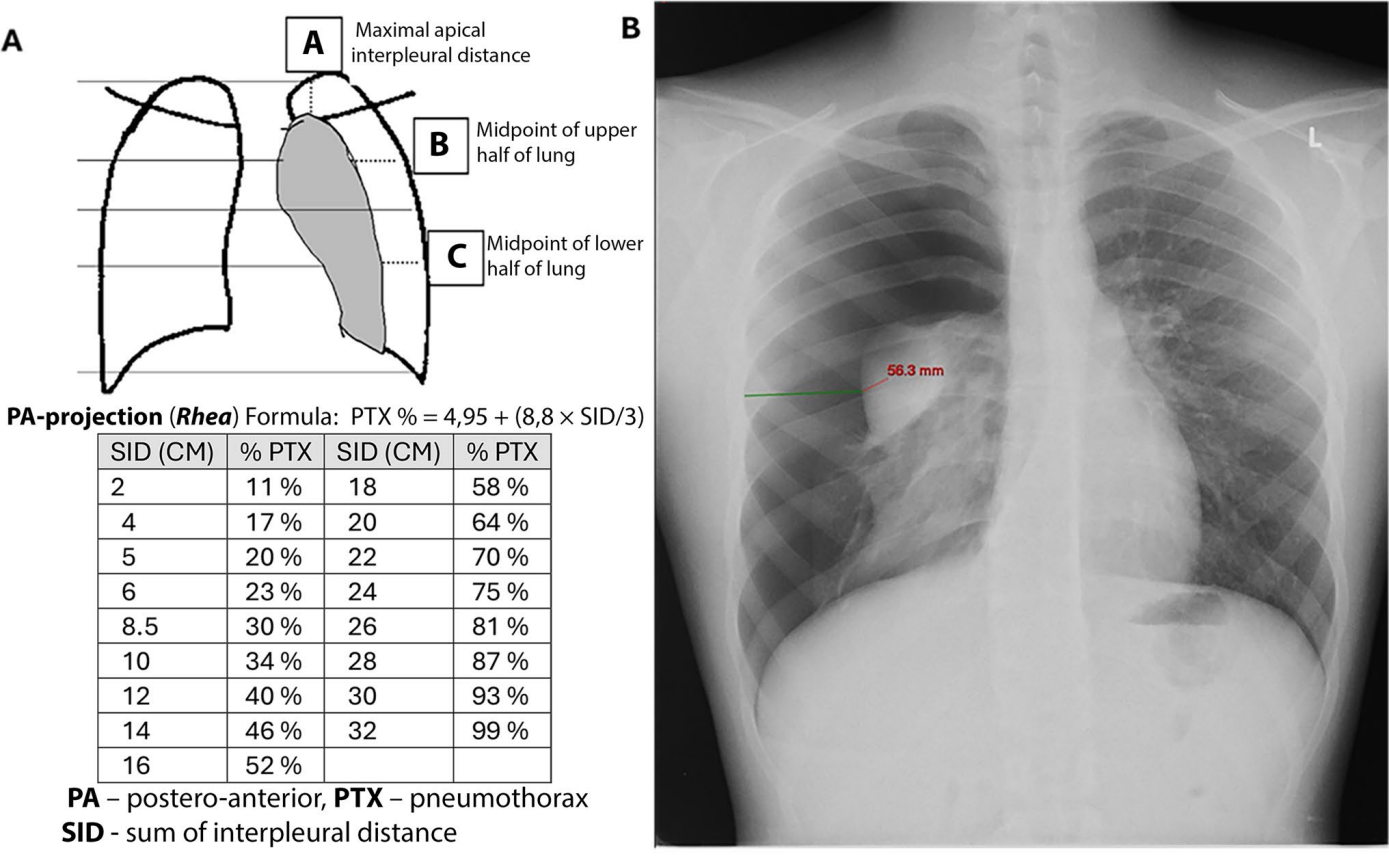
Rhea method for estimating pneumothorax size. demonstration of the Rhea method for estimating pneumothorax size. **A** Illustration of the method involves measuring the distance between the visceral pleura and the chest wall at three points: apex of the lung, midpoint of the upper lung, and midpoint of the lower lung. **B** Displaying a patient’s chest X-ray measurement. The green line represents the midpoint of the upper lung

The pneumothorax size was estimated using the Rhea method [6]. This method involves measuring the average distance between the visceral pleura and the chest wall at three points: the apex of the lung, the midpoint of the upper lung, and the midpoint of the lower lung. The average of these measurements was then correlated with the percentage tables to determine the size of the pneumothorax (Fig. 1) [7, 8]. The size of the pneumothorax was classified as small (<30% of the hemithorax) or large (>30% of the hemithorax). The presence of any radiological findings was considered a positive X-ray sign. These included apical irregularity with excess lucencies, apical blebs or bullae, a visible visceral pleural line, absence of peripheral lung markings, pulmonary collapse with or without mediastinal shift, and signs of radiologic tension pneumothorax. Radiologic tension pneumothorax was defined as a pneumothorax with contralateral mediastinal shift.

#### Chest computed tomography (chest CT)

All available CT scans were reviewed by a single radiologist and independently verified by two other radiologists. Under blinded settings, CT measurements included the presence of blebs and bullae. Blebs were described as air-filled lesions less than 1 cm in diameter, while bullae were more than 1 cm. Furthermore, the correlation between Chest X-Ray (CXR) and CT findings was analyzed.

### Statistical analysis

Categorical variables were reported as frequencies and percentages, while continuous variables were expressed as means ± standard deviations or medians with ranges, based on distribution. Comparisons between operated and non-operated groups were made using chi-square tests for categorical data and *t*-tests or Mann-Whitney *U* tests for continuous data. A logistic regression model was used to identify factors associated with surgical intervention. Variables with *p* < 0.1 in univariate analysis were included in the multivariate model. Model fit was evaluated with the Hosmer-Lemeshow test, and explanatory power was assessed using Cox & Snell and Nagelkerke *R* square values.

All statistical tests were two-sided, with a significance level set at 0.05. Odds ratios (OR) with 95% confidence intervals (CI) were calculated for the logistic regression model. Statistical analyses were performed using SPSS version 29.

## Results

A total of 234 patients were identified with a diagnosis of PSP during the study period. Of these, 91 had imaging available for review and were included in the study. Fifty-six (61.5%) cases underwent non-operative management and 35 (38.5%) cases were operated on (Table 1). Of the 35 patients who were operated on, 9 patients were operated on during their first episode due to prolonged air leak (range 7–13 days). Smoking status was similar between the non-operated group (16.1%) and the operated group (14.3%) (*P* = 0.453). The median systolic blood pressure was similar between groups. The median diastolic blood pressure was significantly higher in the group that underwent surgery compared to the group that did not undergo surgery (75 mmHg vs. 69 mmHg, *P* = 0.023). There were no significant differences in the median heart rate and O_2_ saturation values between the two groups (*P* = 0.208 and *P* = 0.970, respectively). No clinical tension pneumothorax cases were recorded.

**Table 1:** Patient demographics, pneumothorax presentation, imaging characteristics, and treatment outcomes in relation to surgical intervention^a^

	Total *N* = 91 (%)	Operated *N* = 35 (%)	Non-Operated *N* = 56 (%)	*P* value
Patient demographics and baseline characteristic				
Smoker, *N* (%)	14 (15.4)	5 (14.3)	9 (16.1)	0.453
Patient metrics, median (range)				
Systolic blood pressure	118 (67)	120 (31)	117 (67)	0.197
Diastolic blood pressure	70 (64)	75 (29)	69 (64)	**0.023**
Pulse	73 (63)	77 (47)	72 (63)	0.208
Saturation	99 (28)	99 (6)	99 (28)	0.970
Pneumothorax presentation, *N* (%)				
Duration of symptoms				0.100
Day of admission	50 (55)	17 (48.5)	33 (58.9)	
One day before admission	17 (18.7)	7 (20)	10 (17.8)	
2–7 days	20 (22)	9 (25.7)	11 (19.6)	
14–30 days	2 (2)	1 (2)	1 (2)	
Missing data	1 (1)	1 (2)	0	
Large pneumothorax	49 (53.8)	11 (31.4)	38 (67.9)	0.952
Rhea number (median (range))	20 (85)	20 (75)	20 (85)	0.822
Recurring event	35 (38.5)	26 (74.3)	9 (16.1)	**<0.0001**
Imaging characteristics, *N* (%)				
Number of X-rays	5 (16)	5 (10)	5 (16)	0.305
Positive X-ray signs	62 (68.1)	15 (42.9)	47 (83.9)	0.628
Apical irregularity with excess lucencies	57 (62.6)	14 (40.0)	43 (76.8)	0.521
Pulmonary collapse with/without apical irregularity	4 (4.4)	0 (0.0)	4 (7.1)	
No findings	23 (25.3)	5 (14.3)	18 (32.1)	
CT scans	18 (19.8)	8 (22.9)	10 (17.9)	0.017
CT matches X-ray	17 (18.7)	7 (2.3)	10 (17.9)	0.811
Tension pneumothorax^b^	13 (14.3)	3 (8.6)	10 (17.9)	0.946
Treatment and outcomes, *N* (%)				
Drain type				0.06
Big	16 (17.5)	8 (22.8)	8 (14.2)	
Small	49 (53.8)	16 (45.7)	33 (58.9)	
Conservative	18 (19.7)	4 (11.4)	14 (25)	
Missing data of a previous event	8 (8.7)	7 (20)	1 (1.7)	
Drainage effect				
The lung expanded	66 (72.5)	16 (45.7)	50 (89.3)	0.553
Fast improvement	54 (59.3)	13 (37.1)	41 (73.2)	0.688

*CT* computerized tomography

^a^Data was unavailable for some of the patients in several rows

^b^Radiological tension pneumothorax. No clinical tension pneumothorax was recorded in our study

Most patients who presented on the day symptoms began were treated non-operatively (33/56, 59%), compared to those who underwent surgical intervention but with no statistical significance (17/35, 48%, *P*=0.98). In contrast, a higher proportion of operations were performed on patients who had symptoms at least 1 day before admission, but there was no significant difference between the groups based on symptom duration (7/56, 20% non-operated vs. 10/35, 17.8% operated, *P*=0.100).

The prevalence of large pneumothorax was similar between both the non-operated (67.9%) and operated (31.4%) groups (*P* = 0.952). Thirty-five (38.5%) of the cases in the study had a history of pneumothorax and had a reoccurring pneumothorax event. These individuals were substantially more likely to require surgical intervention (*P* < 0.0001). Among the 35 patients with recurrence, 26 (74.3%) had surgery, whereas only 9 (25.7%) were treated non-operatively, *P*<0.0001. All operated patients underwent video-assisted thoracoscopic surgery (VATS) with apical wedge resection and mechanical pleurodesis. Of the 35 patients who were operated on, 7 (20%) patients were operated on due to persistent air leak.

A total of 62 patients (68.1%) exhibited positive X-ray findings. Positive chest X-ray signs were observed in 83.9% of the non-operated group and 42.9% of the operated group, with no significant difference between the groups (*P* = 0.628). These signs included apical irregularity with excess lucencies, present in 76.8% of non-operated patients and 40.0% of operated patients (*P* = 0.521). Pulmonary collapse with or without apical irregularity was rare, found only in the non-operated group (7.1%). Radiologic tension pneumothorax was seen in 17.9% of the non-operative group and 8.6% of the operative group, with no statistically significant difference between the groups (*P* = 0.946).

Of the 91 patients who were included in the study, 18 (19.8%) underwent CT scans, all of them demonstrating findings suggestive of apical blebs (*N*=16) or bullae (*N*=2). Twelve CT scans (66%) were plain and 6 (33%) were contrast enhanced. A higher rate of patients underwent CT scans among the surgical group (22.9% vs. 17.9%, *P*=0.017). A strong correlation between CT and chest radiograph findings was observed in 17 (89.5%) cases of the patients who underwent both CXR and a CT scan (kappa’s score 0.944, 95% CI 0.83–1.00).

Drain type showed a trend towards smaller drains or conservative, non-drain management in non-operated patients (*P* = 0.06). Complete lung expansion following drainage was more prevalent among the non-operated group (89.3% vs. 45.7%, *P* = 0.553). Fast improvement was observed at a higher rate among non-operated patients (73.2% vs. 37.1%, *P* = 0.688).

Table 2 shows the logistic regression analysis used to predict surgical intervention. The presence of positive X-ray signs did not significantly predict surgical intervention (OR = 0.920, 95% CI 0.239–3.533, *P* = 0.903) as well as the type of drain (*P* = 0.137). However, patients who experienced symptoms 1 day before admission had a 4.122-fold higher likelihood of undergoing surgery than those whose symptoms started on the day of admission (95% CI 1.004–19.92, *P* = 0.049).

**Table 2:** Logistic regression analysis of factors predicting surgical intervention in primary spontaneous pneumothorax patients

	OR	CI 95% Lower	Upper	*P* value
X-ray signs	0.920	0.239	3.533	0.903
Drain type (size)				0.137
Small	0.286	0.076	1.085	0.066
Conservative	0.153	0.015	1.927	0.252
Duration of symptoms (day of admission)				0.252
One day before admission	4.122	1.004	19.920	0.049
2–7 days	2.288	0.524	9.986	0.271
14–30 days	2.556	0.123	53.296	0.295

*OR* odds ratio, *CI* confidence interval

## Discussion

The purpose of this study was to identify predictors of surgical intervention in PSP, with a focus on radiographic findings and the role of CT scanning. Contrary to our hypothesis, X-ray findings were not significantly associated with the need for surgery. Indeed, a history of pneumothorax (i.e., recurrent events) and longer symptom duration—both well-recognized indications—were significantly linked to surgical intervention. In addition, smaller drain sizes or conservative management were more common in non-operated patients, though this did not reach statistical significance (*P* = 0.060).

Our study found that positive chest X-ray signs were more prevalent in non-operated patients (83.9%) than in operated patients (42.9%), a finding that is consistent with previous studies reporting no significant demographic or radiological differences between patients who underwent surgical intervention and those who did not [9]. Similarly, the size of the pneumothorax determined by X-ray did not predict the need for surgery (*P* = 0.952). While some studies suggest that very extensive pneumothorax (e.g., ≥50% of the hemithorax) and a smoking history warrant surgical intervention [10, 11], our data indicate that chest X-rays, although essential for initial diagnosis and risk assessment, are insufficient as standalone predictors.

The literature also suggests that the presence of bullae or blebs on imaging may serve as an independent risk factor for surgical intervention [8]. Warner et al. [12] showed that the presence and size of apical blebs on CT were associated with recurrence and the need for thoracostomy, while Casali et al. [13] found CT-detected blebs/bullae to be the sole significant predictor of recurrence in multivariate analysis (hazard ratio = 18). Similarly, Cardillo et al. [14] and Sihoe et al. [15] developed CT criteria to identify patients who might benefit from early surgery, with Olesen et al. [16] even advocating for surgery in cases with bullae ≥2 cm on high-resolution CT. Conversely, several investigations [17, 18] reported no correlation between CT-detected bullae and recurrence rates. In our study, operated patients were more likely to have undergone CT scanning—presumably as part of surgical planning—and CT findings correlated closely with X-ray results, suggesting that CT may add little additional information, particularly in young patients.

We also examined the influence of chest drain type on surgical outcomes. Smaller chest drains or conservative management were more common in non-operated patients (*P* = 0.060). Although literature supports the use of small-bore drains for stable patients due to fewer complications and reduced pain compared to larger drains [19, 20], some studies report no significant differences between small and large drains in preventing surgical intervention [21]. It is notable that our increasing use of small-bore, especially pigtail drains, in recent years may have influenced these results.

Interestingly, the operative group exhibited a significantly higher diastolic blood pressure compared to the non-operative group. Although little research links blood pressure to PSP surgery, elevated diastolic pressure in more severe cases may result from compensatory sympathetic activation, pain, anxiety, or even early mediastinal compression in tension pneumothorax [22–24]. However, the minor difference observed likely has limited clinical significance.

While univariate analysis did not reveal a significant relationship between symptom duration and surgical intervention, logistic regression showed that patients whose symptoms began at least 1 day before admission were four times more likely to require surgery than those with same-day onset (*P* = 0.049). This finding aligns with previous studies emphasizing that timely presentation to the emergency department is crucial for effective pneumothorax management [25, 26]. Nevertheless, the model’s explanatory power was modest (Cox & Snell *R*^2^ = 0.110; Nagelkerke *R*^2^ = 0.171).

Finally, our results confirm that a history of pneumothorax is a strong predictor of surgical intervention (*P* < 0.0001), consistent with previous literature [27, 28]. Clinical guidelines from the British Thoracic Society, along with other studies, advocate for surgical intervention in recurrent cases to significantly reduce the risk of further recurrences [29, 30].

## Limitations

This study has several limitations. Its retrospective design may introduce selection bias and limit control over confounding variables. The single-center nature of the study may affect the generalizability of the results to other populations or healthcare settings. As a retrospective, single-center study, our sample size may limit the precision of our estimates and may not detect smaller, but clinically meaningful, differences. Additionally, the study relied on radiological interpretations that, despite efforts to standardize, may be subject to inter-observer variability. Moreover, the modest explanatory power of the logistic regression model (Cox & Snell *R*^2^ = 0.110; Nagelkerke *R*^2^ = 0.171) indicates that only a fraction of the variance was accounted for, underscoring the influence of unmeasured factors such as surgeon and patient preferences, local protocols, and comorbidities. The relatively low R-squared value of the logistic regression model suggests that other unmeasured factors may play important roles in predicting surgical intervention. Moreover, the extended study period (2013–2024) may have introduced variability in imaging protocols, equipment, and clinical management practices. Future prospective, multi-center studies with larger sample sizes are needed to validate and expand upon these findings.

## Conclusions

Our findings indicate that while chest X-rays are essential for the initial diagnosis of PSP, they do not reliably predict the need for surgical intervention. Prolonged symptom duration, mainly 1 day before admission, and a history of recurrence are key determinants of surgery. The strong correlation between X-ray and CT findings suggests that routine preoperative CT adds little value—especially in young patients—and may be unnecessary. Further prospective studies are needed to validate these results and refine imaging strategies in PSP management.

## Supplementary information

ESM 1 (MP4 8014 kb)

## Data Availability

The datasets generated and analyzed during the current study are not publicly available due to institutional and patient confidentiality policies.
